# Implementation, effectiveness, and barriers of obstetric triage in reducing waiting time: a scoping review

**DOI:** 10.1186/s12978-025-01982-7

**Published:** 2025-03-21

**Authors:** Zemenu Yohannes Kassa, Abel F. Dadi, Habtamu Mellie Bizuayehu, Subash Thapa, Getiye Dejenu Kibret, Tahir A. Hassen, Abdulbasit Seid, Daniel Bekele Ketema, Meless G. Bore, Teketo Kassaw Tegegne, Daniel Bogale Odo, Erkihun Amsalu, Sewunet Admasu Belachew, Desalegn Markos Shifti, Kedir Y. Ahmed

**Affiliations:** 1 Rural Health Research Institute, Charles Sturt University, Orange, NSW Australia; 2 College of Medicine and Health Sciences, Hawassa University, Hawassa, Ethiopia; 3 Menzies School of Health Research, Charles Darwin University, Casuarina, NT Australia; 4 Addis Continental Institute of Public Health, Addis Ababa, Ethiopia; 5 First Nations Cancer and Wellbeing (FNCW) Research Program, School of Public Health, The University of Queensland, QLD Brisbane, Australia; 6 Centre for Health Systems and Safety Research, Australian Institute of Health Innovation, Faculty of Medicine, Health and Human Sciences, Macquarie University, Sydney, NSW Australia; 7 Center for Women’s Health Research, College of Health, Medicine and Wellbeing, The University of Newcastle, Newcastle, NSW Australia; 8 School of Public Health and Preventive Medicine, Monash University, Melbourne, VIC Australia; 9 School of Public Health, College of Medicine and Health Science, Debre Markos University, Debre Markos, Ethiopia; 10 The George Institute for Global Health, University of New South Wales (UNSW), Sydney, Australia; 11 School of Nursing and Midwifery, University of Technology Sydney, Sydney, NSW Australia; 12 Institute for Physical Activity and Nutrition, Deakin University, Geelong, VIC Australia; 13 National Centre for Aboriginal and Torres Strait Islander Wellbeing Research, National Centre for Epidemiology and Population Health, Australian National University, Canberra, ACT Australia; 14 Sydney Medical School, Faculty of Medicine and Health, University of Sydney, Sydney, Australia; 15 First Nations Cancer and Wellbeing (FNCW) Research Program, School of Public Health, The University of Queensland, Brisbane, QLD Australia; 16 Translational Health Research Institute, Western Sydney University, Campbelltown Campus, Sydney, Australia

**Keywords:** Obstetric triage, Pregnancy, Childbirth

## Abstract

**Background:**

The assessment of a pregnant woman typically begins at obstetric triage, where healthcare providers evaluate whether life-altering decisions are necessary for the woman and her unborn baby. This scoping review aimed to assess the lack of comprehensive evaluation of across diverse settings of the evidence on the effectiveness, implementation, and barriers to the successful implementation of obstetric triage during pregnancy and childbirth.

**Methods:**

The Arksey and O’Malley scoping review methodological framework and Joanna Briggs Institute Reviewers’ Manual were applied to conduct the scoping review. The Population, Concept, and Context strategy (PCC) was used to develop the review questions, eligibility criteria, and research strategy, incorporating findings from both quantitative and qualitative research. Preferred Reporting Items for Systematic Reviews and Meta-analyses: Extension for Scoping review (PRISMA-ScR) was implemented. A scoping review search was conducted using four databases by specific key words for example: “pregnant woman” OR “postnatal woman” AND “triage” OR “obstetric emergency service” OR “health facility” AND “delivery” OR “childbirth” OR “obstetric” OR “prenatal care” OR “parturition” OR “pregnancy” OR “maternal health services” OR “perinatal care” OR “postnatal care”. Further additional studies or references were culled from included primary studies to identify relevant studies that were missed in the initial search.

**Results:**

The search strategy generated an initial list of 622 studies of which 15 studies were included. The findings revealed that the implementation of obstetric triage can substantially reduce delays in getting care (delay 3) during birth. The barriers within the department which hindered the successful implementation of obstetric triage included shortages of staff and space, burnout and fatigue among health professionals, inadequate knowledge, resistance to change, lack of commitment and responsibility, unclear task descriptions, insufficient supplies, and deficient communications system.

**Conclusion:**

Our findings underscore that the effective implementation of triage was linked to reduced costs, such as decreased waiting times for women, across six countries. However, identified factors frequently hampered the successful implementation of obstetric triage during pregnancy and childbirth. Given that implementing obstetric triage can substantially reduce delays in getting care during pregnancy and childbirth, linked to reducing costs, and the identified barriers need to be addressed.

## Background

The triage is a preliminary clinical assessment process for patients upon their arrival at health facilities and sorts them before a complete diagnosis and treatment [1]. This practice becomes essential during times of overcrowded obstetric emergency departments, where health facilities with resource constraints can only allocate limited medical supplies and unmet needs coupled with a rushed environment [2].

In response to the essential need for a triage system in emergency medicine, numerous countries have launched obstetric triage systems. For example, the United Kingdom (UK) has introduced the Birmingham symptom-specific obstetric triage system (BSOTS) [3], while the United States of America (USA) implements the Maternal Fetal Triage Index (MFTI) [4]. In Canada, the Obstetric Triage Acuity Scale (OTAS) is used [5], and Australia has implemented Obstetric Triage Decision Aid (ODTA) [6]. Switzerland has added obstetric triage to the Swiss Emergency Triage Scale (SETS) [2], and Iran has applied the Iranian Obstetric Triage Index (IOTI) [7], and it is worth considering adding data from low and middle-income countries (LMICs) to address the lack of accepted global standards in this area. Although improvements in obstetric triage systems within individual countries are encouraging, a well-accepted global standard has not yet been developed. This signals relatively less attention, low priority and potentially fewer regulations [8].

Plain language summary

Effective implementation of obstetric triage is crucial in reducing delays in getting care during pregnancy and childbirth, thereby decreasing the risk of adverse obstetric outcomes. This scoping review aimed to explore the lack of comprehensive evaluation of across diverse settings of evidence on the effectiveness, implementation, and barriers to the successful implementation of obstetric triage during pregnancy and childbirth. A delay in getting care during pregnancy and childbirth significantly increased the rates of morbidity and mortality among both newborns and women. Obstetric triage in healthcare settings involves rapidly evaluating a woman's condition upon arrival to assess the severity of the situation and the urgency of the care required. However, there is a paucity of evidence synthesising the effectiveness of implementing obstetric triage in reducing waiting times during pregnancy and childbirth.

Obstetric triage in health facilities involves quickly assessing a woman’s condition upon arrival to determine the woman’s acuity and the urgency of care needed [9], using either a 5-level scoring system [10] or a traffic light system, where a score of 1 indicates the highest priority and a score of 5 represents the lowest priority. A traffic light system is used with ‘red’ showing the highest priority and ‘green’ indicating the lowest priority, providing a clear indication of the urgency of care required for a woman [11]. These interventions are crucial for saving the lives of women and newborns, requiring healthcare providers to make rapid [12], critical decisions that can have lasting impacts [13]. The process ensures that the necessary life-altering decisions are made early to enhance the safety and quality of obstetric care [14].

The obstetric triage system is an emerging concept that has undergone implementation research in numerous health facilities across various countries [15]. This initiative has led to the scaling up of the system, tailored to the specific setups of individual countries, thereby minimising unnecessary delays and interventions during childbirth [10]. The delays in diagnosis and treatment within obstetric triage can be assessed within 10 min [16], and in some cases, within 15 min based on the guidelines of each country’s obstetric triage system [3]. This approach ultimately contributes to optimal birth outcomes by ensuring timely care and appropriate medical decisions [13].

Previous studies focused on triage systems were limited to LMICs [17]. Additionally, their findings exhibit inconsistencies, inconclusive results, that lead to lack of context-specific recommendations. For instance, a study across four countries showed some used a 10-min obstetric triage assessment while others opted for a 15-min assessment [3, 16].

Obstetric triage system implementation varies globally, with studies like one in Ghana demonstrating improved waiting times, diagnostic accuracy, and quality of care despite resource constraints [18]. However, barriers for example, limited resources, insufficient staff training, and integration into existing workflows remains challenges in low-, middle- and high-income countries [18, 19]. Addressing these issues requires a tailored context-specific approach while leveraging global best practices. Therefore, this scoping review aimed to assess the lack of comprehensive evaluation of across diverse settings of the evidence on the effectiveness, implementation, and barriers to the successful implementation of obstetric triage during pregnancy and childbirth.

### Objective

This scoping review has three main objectives, first identify and map the evidence on the implementation of obstetric triage systems in various settings, in second assess the effectiveness of obstetric triage systems in reducing waiting times during pregnancy and childbirth, and third explore enablers and barriers to the implementation of obstetric triage system.

## Methods

This scoping review was applied in line with Joanna Briggs Institute Reviewers’ Manual and Preferred Reporting Items for Systematic Reviews and Meta-analyses: Extension for Scoping review (PRISMA-ScR) [20, 21].The review protocol followed the Preferred Reporting Items for Systematic Reviews and Meta-analyses: Extension for Scoping review (PRISMA-ScR) [21] to report evidence. The Arksey and O’Malley scoping review five stage methodological framework and Joanna Briggs Institute Reviewers’ Manual were used to conduct the present scoping review [20, 22]: (1) identifying the research questions; (2) identifying relevant studies; (3) selecting the studies according to inclusion criteria; (4) charting the data; and (5) summarising and reporting the findings [22].

Identifying the research questions: the research questions of the present scoping review on the lack of comprehensive evaluation of across diverse settings of the evidence on the effectiveness, implementation, and barriers to the successful implementation of obstetric triage during pregnancy and childbirth.

What is the effectiveness, implementation, and barriers to the successful implementation of obstetric triage during pregnancy and childbirth?

### Search strategy

Articles were retrieved from the following electronic bibliographic databases: PubMed/Medline, Embase (Ovid), Maternity and Infant Care and CINAHL for quantitative, and qualitative studies, published in English up to February 20, 2024. In addition, reference research was applied by reviewing selected studies to identify relevant research that was not initially found in the initial search. Boolean operators were applied to combine both Medical Subject Heading (MeSH) and free text search terms, with the search strategy incorporating truncations with the following keywords: “pregnant woman” OR “postnatal woman” AND “triage” OR “obstetric emergency service” OR “health facility” AND “delivery” OR “childbirth” OR “obstetric” OR “prenatal care” OR “parturition” OR “pregnancy” OR “maternal health services” OR “perinatal care” OR “postnatal care”: the initial search strategy, developed from PubMed, was adapted for use in other databases. No limit was set on the publication years and geography area. Restrictions were applied to include only the studies published in English and those involving human participants.

### Eligibility of studies

The inclusion criteria for selection of studies was framed using population, concept, and context (PCC) framework [20]. The details are presented in Table 1.

**Table 1:** Search terms

Population	Pregnant women, postnatal women
Concept	Triage, obstetric emergency service, health facility
Context	Delivery, childbirth, obstetric, prenatal care, parturition, pregnancy, maternal health services, perinatal care, postnatal care

In addition, this scoping review included quantitative, qualitative, and mixed-methods studies focused on obstetric triage during pregnancy and childbirth.

### Exclusion criteria

Studies involving modelling on obstetric triage, review, tool validation, case reports, editorials, commentaries, abstract and conference proceedings, press releases, and studies with methodological flaws were excluded.

### Study selection

**Fig. 1:**
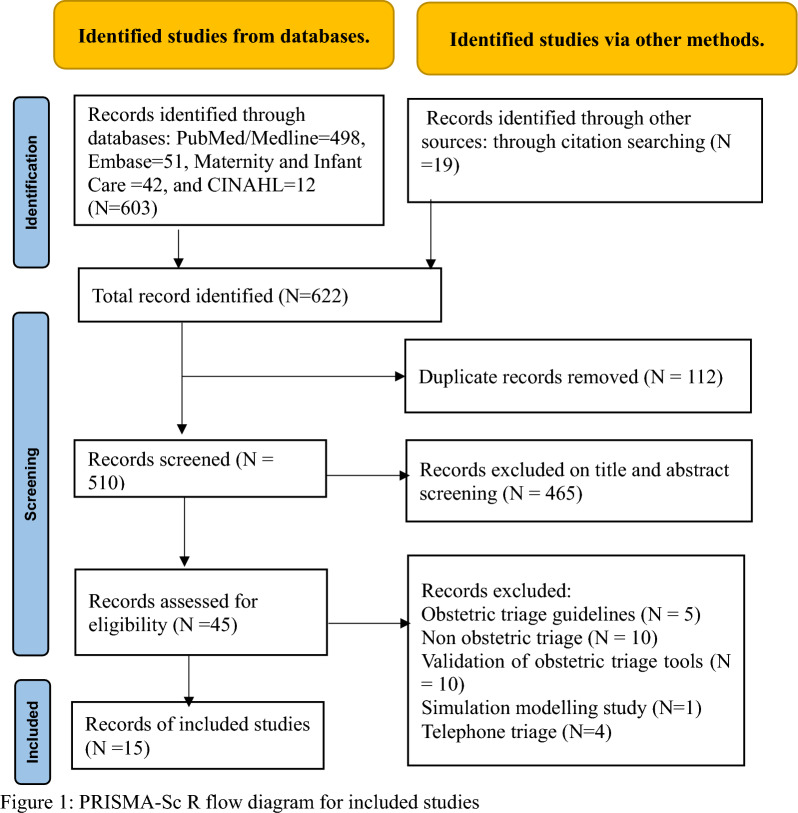
PRISMA-Sc R flow diagram for included studies

The retrieved articles were exported to Endnote 20, where duplicates were removed. Subsequently, the reviewers (ZYK and MG) independently screened identified studies based on their titles and abstracts using predefined inclusion and exclusion criteria. All stages of the inclusion and exclusion procedures applied during the study selection process are presented in the flow diagram, using the PRISMA-ScR guidelines [21] (Fig. 1).

### Data charting process

The data for the present scoping review were systematically extracted from the selected studies based on pre-established criteria, which aligned with the study's objectives. These criteria included study characteristics, populations, methodologies, and outcomes. After screening, the selected studies were summarised in (Table 2), including the authors’ names, year of publication, population, country, sample size, study designs, effectiveness, implementation, and barriers to the successful implementation of obstetric triage during pregnancy and childbirth. Screening and data charting were conducted interchangeably by researchers. Any disagreement among the reviewers was resolved through discussion.

**Table 2:** Summary of studies included in the systematic review of LMICs and high-income countries, and the main findings

Author, Country	Study design	Study population	Study objective	Summary findings
Tukisi, 2022 [23] South Africa	Qualitative	Nine IDIs with midwives	To explore midwives' experience with using obstetric triage tools during labour admission	• Obstetric triage tools lack adequate space to record vital signs as well as routine examinations (physical, abdominal, and vaginal examinations) and rapid laboratory investigations
Ramaswamy, 2023 [18] Ghana	Quantitative (pre- and post-study)	N = 66 (baseline) N = 104 (post implementation)	To describe and evaluate a theory-based implementation approach to introduce obstetric triage within the hospital	• At baseline, the median waiting time from arrival to assessment was 70.5 min (IQR = 30.0–443.0 min). After baseline assessment, obstetric triage training was given to obstetric care providers in the hospital • Waiting time decreased to 6.0 min (IQR = 3.0–15.0) during active implementation • Waiting time decreased to 5.0 min (IQR = 2.0–10.0) during the sustainment period • At baseline, 18% of women were assessed within 10 min of arrival • At implementation, 64.2% of women were assessed within 10 min of arrival • At sustainment, 84.6% of women were assessed within 10 min of arrival • Accuracy of diagnosis at the end of implementation was 75.7% and improved to 77.9% during the sustainment period • The appropriateness of the care plan improved from 66 to 78.9% during the sustainment period
Goodman, 2017 [25] Ghana	Quantitative	Phase1 N = 926	To assess waiting time on obstetric triage at the hospital	• The median waiting time for assessment was significantly longer at night, 55 min (15–135), compared to the morning, 37 min (11–84) and evening, 30-min (12–84) shifts (P < 0.0001) • A median triage time for women with hypertensive disorder during pregnancy was 34 min (11–171), women with obstetric haemorrhage was 24 min (12–185) (P = 0.0079) • A median triage for women in the first stage of labour was 24 min (10–65), and the second stage of labour was 10 min (IQR=5-32) (P < 0.0001)
Goodman, 2018 [26] Ghana	Quantitative (pre- and post-study)	Phase1 N = 926 Phase 2 N = 162 Phase 3 N = 770 Phase 4 N = 869 Phase 5 N = 542	To assess the impact of an obstetric triage improvement program on reducing hospital-based delay on referral	• The obstetric triage improvement program reduced waiting time by 11 min in phase 2 (P < 0.001) • The obstetric triage improvement program reduced waiting time by 33 min in phase 3 (P = 0.002) • Improvements were sustained in phases 4 and 5 in 35 min of reduction in waiting time compared with baseline (P < 0.001) • Obstetric triage improvement program reduced the median (IQR) women waiting time from hospital arrival to first assessment by a midwife from 40 min (15–100) to 5 min (2–6) (P < 0.001) over the 5-year interventions due to empowered midwives to take responsibility for making an initial diagnosis and management plan since the physicians were often unavailable and provided triage assessment training for obstetric care providers • Care plan documentation was increased from 51% in Phase 1 to 96% in Phase 3 (P < 0.001)
Moudi, 2021 [24] Iran	Qualitative	N = 37 health professionals	To explore factors influencing obstetric triage implementation	• The implementation of obstetric triage was hindered by a range of factors such as shortage of human resources, shortage of physical space, lack of motivation leading to burnout and fatigue among health professionals, inadequate knowledge, resistance to change to establish a new process, non-commitment and lack of responsibility, unclear task description, lack of classification based on acuity, lack of supplies, and deficient communications system within the department
Rashidi, 2020 [27] Iran	Qualitative	N = 21	To explain the challenge of obstetric triage structure	• The important challenges in the structure of obstetric triage were identified as a pattern (work shifts for the triage performer) and standard of triage performer, equipment, physical space, human resources and triage procedure and process
Rashidi, 2020 [28] Iran	Qualitative	N = 19	To explain barriers to the quality of the maternity triage process	• Interaction and communication, coordination of healthcare providers within different hospital units, and a tendency for teamwork affected the quality of the maternity triage process • Similarly, the interaction, communication, and coordination of health care providers with women also affected the quality of the maternity triage process
Forshaw, 2016 [11] Uganda	Quantitative	N = 98	To evaluate the effectiveness of the obstetric triage system in the hospital	• The average length of time from entering the department to assessment was 194 min in obstetric triage • When a midwife was presented at the admission desk, the time from entering the department to assessment was decreased from 194 to 38 min • All stages of the journey took longer at night, 8:00 pm to 8:00 am • Shortage of staff and equipment were increased delay from entering to assessment in obstetric triage
Vasilevski, 2023 [32] Australia	Qualitative and quantitative	N = 660 for quantitative N = 9 for qualitative	To evaluate the implementation of Birmingham symptom-specific obstetric triage system	• Average waiting time for triage assessment was 22 min based on the BSOTS colour (Green, Yellow, Orange, and Red) category • Average time to care from triage was 35 min based on the BSOTS colour category • About 12.3% of women spent 6–15 min in triage, indicated by the green colour • About 16.8% of women spent 6–15 min in triage, indicated by the yellow colour • About 32.4% of women spent 6–15 min in triage, indicated by the orange colour • About 25% of women spent 6–15 min in triage, indicated by the red colour • A retrospective audit showed that time to triage for women pre-implementation audits adhered to auditable standards • A retrospective audit showed that time-to-care outcomes for women pre-implementation audits adhered to auditable standards • Lack of knowledge regarding Birmingham's symptom-specific obstetric triage system was considered as a barrier to the effective flow of care of women through the centre
McCarthy, 2022 [6] Australia	Quantitative	N = 2829	To evaluate the implementation of the obstetric triage decision aid	• Preclinical audit found that a median waiting time from arrival to assessment was 21 min (IQR = 10—42), with about 42% of women being seen by midwives within 15 min in the absence of a triage process. Post-implementation of OTDA, the median waiting time from arrival to triage was 9 min (IQR = 5 −16) The proportion of women who were triaged within 15 min of arrival rose at the rate of 42.0–78.0%
Kenyon, 2017 [3] UK	Quantitative	N = 992 FGD (N = 12)	To design, implement and evaluate obstetric triage system	• An increase in the number of women seen within 15 min of attendance from 39% before implementation to 54% post-implementation (RR = 95%CI;1.4 (1.2–1.7) (P < 0.0001) • After implementing the obstetric triage system, midwives indicated an improvement in the safety of women and babies
Kodama, 2021 [29] USA	Quantitative	N = 2679 (1305 historical cohort and 1374 study cohort)	To examine the duration of labour and delivery triage evaluation before and after the implementation of the Maternal Fetal Triage Index	• The implementation of the Maternal Fetal Triage Index showed that the duration of labour and delivery triage evaluation was longer for the study cohort than for the historical cohort (64 min vs 61 min; P = 0.02) • According to the Maternal Fetal Triage Index, women with high priority had a shorter labour and delivery triage evaluation (priority 1, 57 min; priority 2, 66 min; priority 3, 63 min; priority 4, 62 min; and priority 5, 84 min; P < 0.001) • The admission rates were higher in priority 1 and 5 groups (priority 1, 89.3%; priority 2, 41.4%; priority 3, 57.3%; priority 4, 53.3%; and priority 5, 92.6%; P < 0.001)
Smithson, 2016 [1] Canada	Quantitative (pr-and post study)	N = 8085 (obstetric triage visits) pre-implementation N = 8131(obstetric triage visits) post-implementation	To assess the impact of standardised five-category OTAS	• The overall median length of stay for women decreased by 3.9% (P = 0.04) from 105 min (IQR = 52–178) baseline to 101 min (IQR = 49–175)
Quaile, 2018 [31] USA	Quantitative (pr-and post-study)	N = 44 Pre-implementation N = 38 post- implementation	To implement MFTI into maternity units	• Women's average waiting times decreased from 19 min at the baseline to 10.4 during post-implementation
Hoffmann, 2022 [30] USA	Quantitative (pr-and post-study)	N = 370 Pre-implementation N = 254 post- implementation	To assess the impact of MFTI on the management of women presenting severe preeclampsia	• After MFTI was implemented, the median waiting times decreased from 44 min (IQR = 0–65) in epoch one to 17 min (IQR = 0–39) in epoch two (P < 0.001)

### Summarising and reporting the finings

Following data extraction, the findings were summarised by two reviewers (ZYK and MG) simultaneously and any disputes among them were settled with the consultancy of the third reviewer's (EA) for the further analysis process, which is presented in (Table 2). The findings described the characteristics of the included study design, sample, setting, and results, which are the types of studies and objectives of included studies. A narrative synthesis also described women’s waiting times from hospital arrival to first assessment.

### Study outcome

The first outcome of interest of this review was median waiting time related to implementation of obstetric triage. Obstetric triage is a process to comprehensively collect clinical data about a woman and unborn baby upon their arrival within 15 min [23]. The second outcome of interest this review was limited resources, inadequate training, or insufficient staff training, lack adequate space to record vital, shortage of human resources, shortage of physical space, lack of classification based on acuity, and unclear task description [24].

## Results

Of 622 studies retrieved in the database search, 112 duplicates were removed. Subsequently, 465 studies were excluded based on the title and abstract screening. Following full-text review, an additional 30 studies were excluded, leaving 15 studies for data extraction. The selection and screening process is illustrated in Fig. 1.

### Characteristics of included studies

The included studies were conducted in various countries, including LMICs and high-income countries. Specifically, three studies were conducted in Ghana [18, 25, 26], three in Iran [24, 27, 28], three in the USA [29–31], two in Australia [6, 32], and one each in the UK [3], Uganda [11], South Africa [23], and Canada [1]. Ten studies were quantitative (pre- and post-studies), four were qualitative studies, and one combined both quantitative and qualitative methods (Table 2). The study population varied, with the number of IDIs ranging from 9 [23] to 37 [24]. In this study, the sample size of participants ranged from a minimum of 66 at baseline [18] to 1374 participants post-study [29] (Table 2). The included studies were published between 2017 and 2023: two in 2020, [27, 28], two in 2021 [24, 29], two in 2023 [18, 32], and three in 2022 [6, 23, 30] (Table 2).

### Effectiveness of obstetrics triage in reducing median waiting time

Our review demonstrated varying effectiveness of obstetrics triage on reducing median waiting time among the countries. In Ghana, the median waiting time decreased substantially from 70.5 min (IQR = 30.0–443.0) at baseline to 5 min (IQR = 2.0–10.0) during the implementation period [18]. Additionally, the proportion of women assessed within 10 min of arrival increased from 18% at baseline to 64.2% during the implementation period [18]. Similarly, studies have shown that women’s assessment increased from a baseline of 18% to 84.6% within 10 min during the sustainment period in Ghana [18]. Furthermore, the study from Ghana demonstrated that obstetric triage decreased waiting times from a baseline of 40 min (IQR = 15–100) to 5 min (IQR = 2–6) (P < 0.001) over the 5-year interventions in Ghana [26]. Whereas the documentation of care plans increased from 51% in phase one to 96% in phase three (P < 0.001) [26] (Table 2).

In Australia, the median waiting times from arrival to assessment decreased from 21 min (IQR = 10–42) to 9 min (IQR = 5–16) post-implementation [6]. In addition, the proportion of women who triaged within 15 min of arrival increased at the rate between 42.0–78.0% [6]. Similarly, in the UK, there was an increase in the number of women seen within 15 min of attendance, rising from 39% before the implementation of obstetric triage to 54% during the implementation (RR = 95%CI; 1.4 (1.2–1.7), P < 0.0001) [3] (Table 2).

Studies demonstrated that obstetric triage systems and their varying effectiveness between high income countries and LMICs were due to the influence implementation factors. Factors for effective obstetric triage included regular obstetric triage training for healthcare providers, a well-equipped triage system, and sufficient space for the obstetric triage process, which are all dependent on the resources available [18].

In contrast, the barriers hindering obstetric triage included a lack of adequate space for recording vital signs, routine and rapid laboratory investigations [23], shortage of human resources, limited physical space, and low motivation leading to burnout and fatigue among health professionals [24, 27] (Table 2). Other factors included inadequate knowledge, resistance to change to establish a new process, non-commitment, and lack of responsibility [11, 24]. Unclear task description, lack of classification based on acuity, lack of supplies, and deficient communications system within the department further contribute to the challenges affecting the obstetric triage process [24, 28] (Table 2).

## Discussion

The purpose of this scoping review was to synthesise implementation, effectiveness, and barriers of obstetric triage in reducing waiting time. The findings revealed that the implementation of obstetric triage can substantially reduce delays in getting care (delay 3) during pregnancy and childbirth, however the effectiveness differed between the LMICs and the high income countries [18, 19]. The factor for this difference included shortages of staff and space, burnout and fatigue among healthcare providers, inadequate knowledge, resistance to change, lack of commitment and responsibility, unclear task descriptions, insufficient supplies, and deficient communications system within the department hindered successful implementation of obstetric triage. The successful implementation of triage was associated with reduced costs (e.g., waiting time for patients) in 6 countries.

The findings of this scoping review indicate that the implementation of obstetric triage can substantially reduce delays in getting care (delay three) during pregnancy and childbirth, as well as the acuity of care for a woman requiring urgent attention [9]. This aligns with a broader effort to achieve sustainable development goal 3.1 by 2030 [33]. These findings demonstrate that interventions in obstetric triage implementation can significantly lessen delays in assessing a woman upon arrival at health facilities, thereby decreasing the time threshold from 10 min to just 5 min [6, 18, 26]. The standards Association of Women’s Health, Obstetric and Neonatal Nurses (AWHONN) dictate that a woman's vital signs should be assessed within 10 min of arrival [34]. In addition, obstetric triage models such as BSOST, ODTA and MFTI assess a woman within a 10-min of her arrival at the hospital and are used as a threshold [3, 4, 6].

The aforementioned evidence (BSOST, ODTA and MFTI) is translated to practice in LMICs [11, 24, 26] through implementation research, an integral part of evidence-based decision-making efforts that address the existing gaps in translating research evidence into health policy and practice [35]. In this study, interventions in obstetric triage led to an increase in diagnostic accuracy from baseline to during the sustainment period [18]. The implementation and adoption of obstetric triage, especially in LMICs, can decrease maternal and neonatal morbidity and mortality associated with complications of pregnancy and childbirth, including postpartum haemorrhage, pregnancy-induced hypertension and other medical conditions [36]. In addition, a qualitative study in Iran demonstrated that obstetric triage reduced maternal mortality by accelerating care provision at the right time and place for appropriate women [14]. Among its range of benefits, obstetric triage is one of the strategies endorsed and cascaded in health facilities in lowering maternal and neonatal morbidity and mortality, while it is not implemented at the desired level in LMICs [37]. Furthermore, a study demonstrated that in LMICs, women were evaluated in a conventional way based on the time of their arrival. This approach has led to an unbalanced and inequitable approach, resulting in delayed initial assessment, long waiting times, and negatively impacting clinical outcomes [38]. This plausible evidence [25] indicates that introducing implementation research on obstetric triage and scaling up obstetric triage in LMICs is still in its infancy.

These findings identified a range of barriers to implementing obstetric triage. The barrier is the standard of triage performance [27], obstetric triage tools did not provide sufficient space to record vital signs, routine examinations (physical, abdominal and vaginal) and routine laboratory investigations [23], shortage of human resources, lack of physical space, and low motivation, leading to burnout and fatigue among health professionals [24, 27]. Additionally, contributing factors include inadequate knowledge, resistance to change to establish a new process, non-commitment, and lack of responsibility [11, 24]. Unclear task description, lack of classification based on acuity, lack of medical supplies, and lack of coordination and communication system within the department at the hospital [24, 28]. Consequently, these factors result in the diminished provision of optimal obstetric care, leading to increased maternal and neonatal mortality, especially in low-resource settings [39]. The implications of the findings provide a clue on implementation research on obstetric triage in LMICs and how to adopt and adapt the obstetric triage model tailored to a specific health system set up of each country.

This review highlights the paucity of studies assessing implementation research on obstetric triage. This review includes studies that focus on a diverse range of health systems, allowing the synthesis of a comprehensive understanding of the implementation challenges associated with obstetric triage. The resulting synthesis may provide valuable insights into the development of context-specific guidelines in LMICs. However, a variety of limitations need to be acknowledged, as the included studies were only found in 15 middle- and high-income countries. Firstly, it was limited to articles published in English, potentially excluding relevant studies in other languages. Second, the inclusion of studies from specific databases might have led in the exclusion of pertinent grey literature. Furthermore, variability in the conceptualisation of obstetric triage across studies posed challenges for synthesis. Therefore, precautions should be considered when generalising the findings to other middle- and high-income countries. This study lacked data from low-income countries, leading to inequity regarding the translation of evidence into practice on obstetric triage.

## Conclusions

The findings highlight that the implementation of obstetric triage can substantially reduce delays in getting care (delay 3) during pregnancy and childbirth, however the effectiveness differed between the LMICs and the high-income countries. The translation of evidence regarding the implementation of obstetric triage into practice is crucial for reducing delays in getting care during pregnancy and childbirth.

## Data Availability

No datasets were generated or analysed during the current study.

## References

[1] Smithson DS, Twohey R, Watts N, Gratton RJ. The impact of standardized acuity assessment and a fast-track on length of stay in obstetric triage: a quality improvement study. J Perinat Neonatal Nurs. 2016;34(4):310–8. 27513609 10.1097/JPN.0000000000000193

[2] Veit-Rubin N, Brossard P, Gayet-Ageron A, Montandon CY, Simon J, Irion O, et al. Validation of an emergency triage scale for obstetrics and gynaecology: a prospective study. BJOG. 2017;124(12):1867–73. 28294509 10.1111/1471-0528.14535

[3] Kenyon S, Hewison A, Dann SA, Easterbrook J, Hamilton-Giachritsis C, Beckmann A, Johns N. The design and implementation of an obstetric triage system for unscheduled pregnancy related attendances: a mixed methods evaluation. BMC Pregnancy Childbirth. 2017;17(1):309. 28923021 10.1186/s12884-017-1503-5 PMC5604363

[4] Ruhl C, Scheich B, Onokpise B, Bingham D. Content Validity Testing of the maternal fetal triage index. J Obstet Gynecol Neonatal Nurs. 2015;44(6):701–9. 26469714 10.1111/1552-6909.12763

[5] Gratton RJ, Bazaracai N, Cameron I, Watts N, Brayman C, Hancock G, et al. Acuity assessment in obstetrical triage. J Obstet Gynaecol Can. 2016;38(2):125–33. 27032736 10.1016/j.jogc.2015.12.010

[6] McCarthy MF, Pollock WE, McDonald SJ. Implementation of an obstetric triage decision aid into a maternity assessment unit and emergency department. Women Birth. 2022;35(3):e275–85. 34183275 10.1016/j.wombi.2021.06.001

[7] Moudi A, Iravani M, Najafian M, Zareiyan A, Forouzan A, Mirghafourvand M. The development and validation of an obstetric triage acuity index: a mixed-method study. J Matern Fetal Neonatal Med. 2022;35(9):1719–29. 32495659 10.1080/14767058.2020.1768239

[8] Moudi A, Iravani M, Najafian M, Zareiyan A, Forouzan A, Mirghafourvand M. Obstetric triage systems: a systematic review of measurement properties (Clinimetric). BMC Pregnancy Childbirth. 2020;20(1):275. 32375808 10.1186/s12884-020-02974-0 PMC7203833

[9] Ruhl C, Garpiel SJ, Priddy P, Bozeman LL. Obstetric and fetal triage. Semin Perinatol. 2020;44(4): 151240. 32279833 10.1016/j.semperi.2020.151240

[10] Smithson DS, Twohey R, Rice T, Watts N, Fernandes CM, Gratton RJ. Implementing an obstetric triage acuity scale: interrater reliability and patient flow analysis. Am J Obstet Gynecol. 2013;209(4):287–93. 23535239 10.1016/j.ajog.2013.03.031

[11] Forshaw J, Raybould S, Lewis E, Muyingo M, Weeks A, Reed K, et al. Exploring the third delay: an audit evaluating obstetric triage at Mulago National Referral Hospital. BMC Pregnancy Childbirth. 2016;16(1):300. 27724846 10.1186/s12884-016-1098-2 PMC5057228

[12] Power C, Williams C, Brown A. Does a mother’s childbirth experience influence her perceptions of her baby’s behaviour? A qualitative interview study. PLoS ONE. 2023;18(4): e0284183. 37023064 10.1371/journal.pone.0284183 PMC10079033

[13] Barnea ER, Muller M, Di Simone N, Inversetti A, Pacagnella R, Borovac-Pinheiro A, Nicholson W. Prep-for-Labor: overview of FIGO’s labor and delivery triage bundles of care to optimize maternal and newborn outcomes. Int J Gynaecol Obstet. 2023;163(Suppl 2):34–9. 37807589 10.1002/ijgo.15112

[14] Moudi A, Iravani M, Najafian M, Zareiyan A, Forouzan A, Mirghafourvand M. Exploring the concept and structure of obstetric triage: a qualitative content analysis. BMC Emerg Med. 2020;20(1):74. 32933481 10.1186/s12873-020-00369-0 PMC7493847

[15] Angelini DJ. Obstetric triage and advanced practice nursing. J Perinat Neonatal Nurs. 2000;13(4):1–12. 11075082 10.1097/00005237-200003000-00002

[16] Paisley KS, Wallace R, DuRant PG. The development of an obstetric triage acuity tool. MCN Am J Matern Child Nurs. 2011;36(5):290–6. 21857199 10.1097/NMC.0b013e318226609c

[17] Zachariasse JM, van der Hagen V, Seiger N, Mackway-Jones K, van Veen M, Moll HA. Performance of triage systems in emergency care: a systematic review and meta-analysis. BMJ Open. 2019;9(5): e026471. 31142524 10.1136/bmjopen-2018-026471 PMC6549628

[18] Ramaswamy R, Bogdewic S, Williams CR, Deganus S, Bonzi GA, Boakye J, et al. Implementation matters: assessing the effectiveness and sustainment of an obstetric triage program at a high-volume facility in Ghana. Implement Sci Commun. 2023;4(1):138. 37968768 10.1186/s43058-023-00527-y PMC10647175

[19] Lindroos L, Sengpiel V, Elden H. Experiences of implementing and working with obstetric emergency triage: a qualitative study among Swedish midwifes, auxiliary nurses, and obstetricians. Sex Reprod Healthc. 2024;40: 100958. 38492272 10.1016/j.srhc.2024.100958

[20] Peters MD, Godfrey CM, McInerney P, Soares CB, Khalil H, Parker D. The Joanna Briggs Institute reviewers' manual 2015: methodology for JBI scoping reviews. 2015.

[21] Tricco AC, Lillie E, Zarin W, O’Brien KK, Colquhoun H, Levac D, et al. PRISMA Extension for Scoping Reviews (PRISMA-ScR): Checklist and Explanation. Ann Intern Med. 2018;169(7):467–73. 30178033 10.7326/M18-0850

[22] Arksey H, O’Malley L. Scoping studies: towards a methodological framework. Int J Soc Res Methodol. 2005;8(1):19–32.

[23] Tukisi KP, Temane A, Nolte A. “A tool we need”: midwives’ descriptions and recommendations of an ideal obstetric triage tool. Health SA. 2022;27:2029. 36337445 10.4102/hsag.v27i0.2029 PMC9634682

[24] Moudi A, Iravani M, Najafian M, Zareiyan A, Forouzan A, Mirghafourvand M. Factors influencing the implementation of obstetric triage: a qualitative study. Midwifery. 2021;92: 102878. 33161173 10.1016/j.midw.2020.102878

[25] Goodman DM, Srofenyoh EK, Olufolabi AJ, Kim SM, Owen MD. The third delay: understanding waiting time for obstetric referrals at a large regional hospital in Ghana. BMC Pregnancy Childbirth. 2017;17(1):216. 28693518 10.1186/s12884-017-1407-4 PMC5504760

[26] Goodman DM, Srofenyoh EK, Ramaswamy R, Bryce F, Floyd L, Olufolabi A, et al. Addressing the third delay: implementing a novel obstetric triage system in Ghana. BMJ Glob Health. 2018;3(2): e000623. 29707245 10.1136/bmjgh-2017-000623 PMC5914900

[27] Rashidi Fakari F, Simbar M. Explaining challenges of obstetric triage structure: a qualitative study. Nurs Open. 2020;7(4):1074–80. 32587726 10.1002/nop2.478 PMC7308674

[28] Rashidi-Fakari F, Simbar M, Safari S, Zadeh-Modares S, Alavi-Majd H. The quality of the maternity triage process: a qualitative study. Adv J Emerg Med. 2020;4(1): e6. 31938775 10.22114/ajem.v0i0.242 PMC6955035

[29] Kodama S, Mokhtari NB, Iqbal SN, Kawakita T. Evaluation of the Maternal-Fetal Triage Index in a tertiary care labor and delivery unit. Am J Obstet Gynecol MFM. 2021;3(4): 100351. 33757932 10.1016/j.ajogmf.2021.100351

[30] Hoffmann E, Wilburn-Wren K, Dillon SJ, Barahona A, McIntire DD, Nelson DB. Impact of implementation of the maternal-fetal triage index on patients presenting with severe hypertension. Am J Obstet Gynecol. 2022;227(3):521.e1-e8. 35697094 10.1016/j.ajog.2022.06.006

[31] Quaile H. Implementing an obstetrics-specific triage acuity tool to increase nurses’ knowledge and improve timeliness of care. Nurs Womens Health. 2018;22(4):293–301. 30077235 10.1016/j.nwh.2018.05.002

[32] Vasilevski V, Ryan D, Crowe G, Askern A, McCormick M, Segond S, Sweet L. Evaluating the implementation of the Birmingham symptom-specific obstetric triage system (BSOTS) in Australia. Women Birth. 2023;36(3):290–8. 36127283 10.1016/j.wombi.2022.09.005

[33] Assembly G. Resolution adopted by the general assembly on 11 september 2015. New York: United Nations; 2015.

[34] Tepner L, Yogel D, Chichester M. Ten minutes to increased patient satisfaction in the obstetric triage. J Obstet Gynecol Neonatal Nurs. 2020;49(6):S62.

[35] Coopey M, Nix MP, Clancy CM. Translating research into evidence-based nursing practice and evaluating effectiveness. J Nurs Care Qual. 2006;21(3):195–202. 16816597 10.1097/00001786-200607000-00001

[36] World Health Organisation. WHO Guidelines Approved by the Guidelines Review Committee. Geneva. 2023.

[37] Williams CR, Bogdewic S, Owen MD, Srofenyoh EK, Ramaswamy R. A protocol for evaluating a multi-level implementation theory to scale-up obstetric triage in referral hospitals in Ghana. Implement Sci. 2020;15(1):31. 32398109 10.1186/s13012-020-00992-2 PMC7218616

[38] Naz S, Saleem S, Shamsul Islam Z, Bhamani S, Sheikh L. Obstetric triage improvement process using the Donabedian model of quality care: a quality improvement initiative. BMJ Open Qual. 2022;11(2):e001483. 35577399 10.1136/bmjoq-2021-001483 PMC9115037

[39] Lindroos L, Elden H, Karlsson O, Sengpiel V. An interrater reliability study on the Gothenburg obstetric triage system—a new obstetric triage system. BMC Preg Childbirth. 2021;21(1):668. 10.1186/s12884-021-04136-2 PMC8487102 34600512

